# Dose-volume histogram predictors of chronic gastrointestinal complications after radical hysterectomy and postoperative intensity modulated radiotherapy for early-stage cervical cancer

**DOI:** 10.1186/1471-2407-14-789

**Published:** 2014-10-29

**Authors:** Zhongjie Chen, Li Zhu, Bailin Zhang, Maobin Meng, Zhiyong Yuan, Ping Wang

**Affiliations:** Department of Radiation Oncology, Tianjin Medical University Cancer Institute and Hospital, National Clinical Research Center for Cancer, Key Laboratory of Cancer Prevention and Therapy, Tianjin, China

## Abstract

**Background:**

The small bowel is one of the critical organs involved in gastrointestinal complications in cervical cancer treated with postoperative intensity modulated radiotherapy. Even with modest doses of radiation therapy (45-50Gy), the risk of severe injury from postoperative radiation therapy is between 5% and 15%. Up to now, a predictive model of acute GI complications of the small bowel has been established with the aid of Quantitative Analyses of Normal Tissue Effects in the Clinic. However, the correlation between dose-volume effect and chronic GI complications of the small bowel has not been extensively investigated. In the article, the correlation has been studied preliminarily.

**Methods:**

This study analyzed 84 patients who underwent postoperative IMRT. The organ at risk that was contoured was the small bowel loops. DVH parameters subjected to analysis included maximum and mean dose, the volume of these organs receiving more than 30, 40, and 50 Gy (V30-50 volume) and the volume of V30-50 to total volume (V30-50 ratio). Association between DVH parameters or clinical factors and the incidence of grade 1–2 chronic GI complications were evaluated.

**Results:**

Body position and RT total dose are significantly associated with grade 1–2 chronic GI complications after postoperative IMRT in early-stage cervical cancer patients. Maximum dose and V40 ratio of the small bowel loops were significantly associated with chronic GI complications (*P* < 0.05). The optimal threshold were 5586 cGy (maximum dose) and 28% (V40 ratio) of the small bowel loops.

**Conclusions:**

Maximum dose and V40 ratio of the small bowel loops should be considered synthetically before postoperative IMRT for early-stage cervical cancer.

## Background

Adjuvant whole pelvic radiation therapy (WPRT) after radical hysterectomy reduces locoregional recurrence in cervical cancer patients after surgery with adverse risk factors [1, 2]. Adjuvant concurrent chemoradiation therapy has been shown to improve survival rates for high-risk cervical cancer patients compared with adjuvant WPRT alone [3, 4]. Unfortunately, after hysterectomy, small bowel tends to fall into the vacated space in the true pelvis, increasing the amount of bowel treated to high dose. The small bowel is one of the critical organs involved in gastrointestinal (GI) complications. Even with modest doses of radiation therapy (45-50Gy), the risk of severe injury from postoperative radiation therapy is between 5% and 15% [5, 6]. Up to now, a predictive model of acute GI complications of the small bowel has been established with the aid of Quantitative Analyses of Normal Tissue Effects in the Clinic (QUANTEC) [7]. However, the correlation between dose-volume effect and chronic GI complications of the small bowel in cervical cancer treated with postoperative intensity modulated radiotherapy (IMRT) has not been extensively investigated.

Since 2010, we have been using postoperative IMRT for early-stage cervical cancer patients with adverse risk factors. The purpose of the study reported here was to evaluate dose-volume histogram (DVH) predictors for the development of chronic GI complications in cervical cancer patients who underwent radical hysterectomy and postoperative IMRT.

## Methods

### Patients

A total of 95 patients with cervical cancer received radical hysterectomy and postoperative IMRT at our institute between January 2010 and June 2012. Treatment criteria for postoperative IMRT were indicated according to Adjuvant Treatment in the NCCN Guidelines for cervical cancer [1, 8–11]. 11 of these patients were excluded from the study: 3 who received extended-field radiation therapy because of para-aortic lymph node metastases; 3 who received re-radiotherapy because of pelvic lymph node metastases after the primary radiotherapy; and 5 who died because of distant metastases after postoperative IMRT. The remaining 84 patients treated with radical hysterectomy and postoperative IMRT were analyzed for this study with a minimum follow-up period of 3 months.

All eligible patients were examined in a multidisciplinary setting by surgery (Wang K), medical oncology (Huang D), and radiation oncologist (Chen ZJ, Zhu L, and Zhang BL) before being enrolled into the study. The study protocol was in accordance with the ethical guidelines of the 1995 Declaration of Helsinki and was approved by the independent ethics committees at Tianjin Medical University Cancer Institute & Hospital, National Clinical Research Center for Cancer (No. Ebc2014). The written informed consent was obtained from all participants.

### Radiation therapy and chemotherapy

Six patients were immobilized in the supine position in an immobilization device in the early period, the others were treated on a commercial bellyboard (KHLD-TJ-1200, Beijing, China). The general set-up was to have the patient lay prone on the bellyboard with the iliac crest aligned to lie between the bellyboard hump and the inferior edge of the opening. RT planning CT (Brilliance, Philips, Holland) was performed with 5-mm slices with normal quiet breathing and a full-bladder scan. The CT scan range was from the upper edge of L3 to at least 7 cm below the bottom of the obturator foramen. A commercial treatment planning system (Pinnacle^3^ RTP, Philips, USA) was used to design the radiation fields. The clinical target volume (CTV) comprised a central vaginal CTV and a regional nodal CTV. The former included the proximal vagina and paravaginal tissues and the latter consisted of the common iliac, external and internal iliac, and presacral lymph nodes. CTV were contoured according to the consensus guidelines of the Radiation Therapy Oncology Group (RTOG) 0418 and its atlas on the RTOG website [12]. The planning target volume (PTV) was generated by using 7-mm uniform expansion of the CTV. RT was delivered using 6-megavolt X rays from a linear accelerator (Varian 600C/D, USA). 75 patients (89%) received the whole RT dose as planned (50.4 Gy), 9 patients (11%) received more than 50.4 Gy because of concurrent boost radiation therapy to positive pelvic lymph node region (60Gy). The prescribed RT doses were administered in 28 fractions, 1.8-2.14 Gy/fraction, 5 fractions/week. The prescription dose is the isodose which encompasses at least 97% of the vaginal PTV and nodal PTV. No more than 20% of any PTV will receive > 110% of its prescribed dose. No more than 1% of any PTV will receive < 93% of its prescribed dose. 41 patients were given chemotherapy during the course of IMRT for 5 cycles, of which 12 were given cisplatin (40 mg/m^2^) every week, 29 were given docetaxel (40 mg/m^2^) every week.

### Contouring and evaluation of normal structures

The small bowel loops were contoured on every slice, including 2 cm above the PTV. It includes the volume surrounding loops of small bowel out to the edge of the peritoneum because the bowel may lie within this space at any time throughout the course of treatment. The large bowel, rectum and bladder were excluded from the small bowel loops. DVH parameters subjected to analysis included maximum and mean dose to the small bowel loops, V30-50 ratio and volume of this organ. V30-50 volume means volume receiving more than respective dose, and V30-50 ratio means volume receiving more than respective dose to total volume.

### Follow-up and evaluation of chronic GI complications

The patients were followed up by gynecologic and radiation oncologists on an outpatient basis every month in the first year, every 3 months in the second year, every 6 months in the third to the fifth year. We defined a chronic complication as a GI event that occurred more than 3 months after radiation therapy was started. The severity of the GI complication was classified according to the RTOG/European Organization for Research and Treatment of Cancer Late Radiation Morbidity Score, as follows: grade 0, no complications; grade 1, mild fibrosis, mild cramping bowel, movement 5 times daily; grade 2, moderate diarrhea and colic bowel, movement > 5 times daily; grade 3, obstruction or bleeding, requiring surgery; grade 4, necrosis, perforation fistula. Toxicity data including the grade of GI complications were collected retrospectively through hospitalization and follow-up records.

### Statistical analysis

Associations between selected DVH parameters and the incidence of grade 1–2 were evaluated. The relationships between clinical or DVH parameters and the incidence of chronic GI complications were analyzed with the Mann–Whitney *U* test for quantitative variables and the Fisher exact test for categorical variables. Multivariate analysis using Cox regression models was performed to identify risk factors associated with grade 1–2 chronic GI complications. The mean DVH parameters for the small bowel loops with and without GI complications were compared by Mann–Whitney *U* test. Receiver operating characteristics (ROC) curve analysis of each of the DVH parameters with a *P* value of <0.05 in the univariate analysis was performed to select the most relevant threshold for prediction of grade 1–2 chronic GI complication. The predictive value of each parameter was evaluated based on the area under the ROC curve (AUC). The AUC reflects the ability of the test to distinguish between patients with and without chronic GI toxicity. The optimal threshold for each DVH parameter was defined as the point yielding the minimal value for (1-sensitivity)^2^ + (1-specificity)^2^, which is the point on the ROC curve closest to the upper left-hand corner [13]. A *P* value of <0.05 or a 95% confidence interval not encompassing 1 was considered to be statistically significant. All statistical tests were 2-sided.

## Results

The characteristics of the 84 patients are shown in Table 1. The median follow-up period from the end of radiation therapy was 16 months (range 4–36 months). None of the patients experienced a local or distant recurrence within 3 months. The Eastern Cooperative Oncology Group performance status was 0–1 for all patients. The median age of the patients was 47 years old (range 29–68 years old). The median total dose of docetaxel in 29 patients was 160 mg (range 40-280 mg), cisplatin in 12 patients was 160 mg (range 150-240 mg). 56 patients (67%) had grade 0 chronic GI complications, 22 patients (26%) had grade 1, 6 (7%) had grade 2, and no patient had grade 3 or higher chronic GI complications.

**Table 1:** **Patient and treatment characteristics**

No. (%)	
Age (y)	
Mean ± SD	48 ± 8
T-stage	
T1	63(75)
T2	21(25)
N-stage	
N0	73(87)
N1	11(13)
Histology	
SCC	80(95)
Others	4(5)
Smoking	
None	79(94)
Yes	5(6)
Diabetes	
None	77(92)
Yes	7(8)
BMI (kg/m2)	
Mean	25
SD	±4
Body position	
Prone	78(93)
Supine	6(7)
RT total dose (Gy)	
Mean	51
SD	±3
Concurrent chemotherapy	
None	43(51)
Yes	41(49)

*Abbreviations*: SCC = squamous cell carcinoma; BMI = body mass index; RT = radiation therapy; SD = standard deviation.

The incidence of chronic GI complications was analyzed as a function of clinical factors. Because there were few patients with a history of abdominopelvic surgery among the study population, we did not analyze this factor. The results of univariate analyses are shown in Table 2. Body position, RT total dose and concurrent chemotherapy were significantly associated with grade 1–2 GI complications. Then multivariate analysis was performed with these 3 potential risk factors of chronic GI complications. Of the 3 parameters, body position and RT total dose emerged as independent predictors of chronic GI complications (Table 3).

**Table 2:** **Univariate analysis (Mann–Whitney** ***U*** **test and Fisher exact test) for the development of grade 1–2 chronic GI complications**

	Grade 0	Grade 1-2	
Variable	No.	No.	***P***value
Age (y)			
≤48	31	17	0.815
>48	25	11	
T-stage			
T1	42	21	1.000
T2	14	7	
N-stage			
N0	50	23	0.494
N1	6	5	
Histology			
SCC	53	27	1.000
Others	3	1	
Smoking			
None	51	28	0.164
Yes	5	0	
Diabetes			
None	52	25	0.681
Yes	4	3	
BMI (kg/m2)			
≤25	29	16	0.817
>25	27	12	
Body position			
Prone	56	22	0.001
Supine	0	6	
RT total dose			
50.4 Gy	54	21	0.005
>50.4 Gy	2	7	
Concurrent chemotherapy			
None	34	9	0.020
Yes	22	19	
Chemotherapy regimens			
Cisplatin	7	5	0.703
Docetaxel	15	14	

*Abbreviations*: GI = gastrointestinal; SCC = squamous cell carcinoma; BMI = body mass index; RT = radiation therapy.

**Table 3:** **Multivariate analysis for the development of grade 1–2 chronic GI complications**

Variable	HR (95% CI)	***P***value
Body position	4.120 (1.513-11.217)	0.006
RT total dose	3.183 (1.312-7.720)	0.010
Concurrent chemotherapy	1.748 (0.742-4.120)	0.202

*Abbreviations*: GI = gastrointestinal; HR = hazard ratio; CI = confidence interval; RT = radiation therapy.

The mean DVH parameters of the small bowel loops of patients with and without GI complications are shown in Table 4. Patients with grade 1–2 chronic GI complications had significantly greater maximum dose and V40 ratio in the small bowel loops than did those without chronic GI complications (*P* < 0.05).

**Table 4:** **Comparison of mean DVH parameters of the small bowel loops in patients with and without chronic GI complications (Mann–Whitney** ***U*** **test)**

	Overall	Grade 0	Grade 1-2	***P***value
	Maximum dose (cGy ± SE)			
	5600 ± 257	5552 ± 207	5697 ± 318	0.042
	Mean dose (cGy ± SE)			
	2918 ± 500	2838 ± 448	3077 ± 568	0.080
	Mean ratio ± SE			
V30 ratio	0.47 ± 0.14	0.46 ± 0.12	0.51 ± 0.17	0.301
V40 ratio	0.27 ± 0.12	0.24 ± 0.10	0.32 ± 0.15	0.011
V50 ratio	0.12 ± 0.08	0.11 ± 0.07	0.14 ± 0.08	0.134
	Mean volume ± SE (ml)			
Total volume	1008 ± 399	1041 ± 366	943 ± 458	0.261
V30 volume	460 ± 185	466 ± 173	448 ± 211	0.507
V40 volume	257 ± 125	248 ± 119	275 ± 136	0.423
V50 volume	107 ± 62	101 ± 52	119 ± 77	0.451

*Abbreviations*: DVH = dose-volume histogram; GI = gastrointestinal; SE = standard error; V30-50 volume = volume receiving more than respective dose; V30-50 ratio = volume receiving more than respective dose to total volume.

ROC curve analysis was performed to select the most relevant parameter to identify predictors of grade 1–2 chronic GI complications among DVH parameters with a *P* value of <0.05 in the univariate analysis for the small bowel loops (Table 5).

**Table 5:** **ROC curve analysis for DVH parameters of the small bowel loops in relation to grade 1–2 chronic GI complications**

			Optimal threshold	
	AUC	95% CI	Value	Sensitivity/specificity (%)
Maximum dose	0.637	0.503-0.770	5586 cGy	64.3/67.9
V40 ratio	0.670	0.540-0.800	0.28	67.9/57.1

*Abbreviations*: AUC = area under the ROC curve; CI = confidence interval; DVH = dose-volume histogram; GI = gastrointestinal; ROC = receive operating characteristics; V30-50 volume = volume receiving more than respective dose; V30-50 ratio = volume receiving more than respective dose to total volume.

## Discussion

Many studies have introduced predictive factors potentially associated with chronic GI complications after RT for gynecologic malignancies [14–19]. Our study showed that body position was significantly associated with grade 1–2 GI complications in univariate and multivariate analyses (*P* = 0.006, HR = 4.120). The percentage of grade 1–2 toxicity in prone and supine position were 28% and 100%, respectively. Cranmer-Sarqison reported that the use of a bellyboard with IMRT provides excellent small bowel sparing regardless of planning technique [20]. Hollenhorst also discribed that the mean dose to the small bowel was 52.4% when the bellyboard was used, as compared to a mean dose of 63.1% without the bellyboard [21]. Collectively, these results suggest that the use of a bellyboard with IMRT provides better small bowel sparing.

Our study also showed that RT total dose had a significant association with chronic grade 1–2 GI complications (*P* = 0.010, HR = 3.183). So it is important to select the best DVH parameters to predict the possibility of the incidences of chronic GI toxicity. There are two points to be elaborated here about how to select the best DVH parametes. First, IMRT patients had a lower rate of chronic GI toxicity than that of WPRT patients [22, 23]. Our results also noted that the percentage of the cervical cancer patients with grade 1, 2, and 3 toxicity were as low as 26%, 7%, and 0%, respectively. For this reason, the incidence of grade 1 and 2 chronic GI complications were evaluated jointly. Second, the small bowel loops were contoured according to the consensus guidelines of the Radiation Therapy Oncology Group (RTOG) 0418 because they may lie within the volume surrounding loops of small bowel out to the edge of the peritoneum at any time throughout the course of treatment [22]. Han et al. also emphasized that the dose distribution in the small bowel as observed on CT varies significantly from week to week because of the interfractional variations of small bowel positions [7].

The results of univariate analyses showed that maximum dose and V40 ratio of the small bowel loops had a significant association with chronic GI complications (*P* < 0.05). ROC curve analysis showed AUCs for the above 2 DVH parameters were from 0.632 to 0.670, the optimal threshold were 5586 cGy (maximum dose), 28% (V40 ratio) of small bowel loops (Table 5). These findings suggest that the two parameters may constitute a better predictor of chronic GI complications. Similar results were found in other studies. Isohashi reported that V40 of the small bowel loops emerged as independent predictors of GI complications after postoperative concurrent nedaplatin-based chemoradiation therapy in early-stage cervical cancer patients [23]. Kavanagh noted that late small bowel toxicity is likely related to maximum dose and/or volume threshold parameters, qualitatively [22]. Interestingly, our studies showed that V40 ratio of small bowel loops had a significant association with chronic GI complications, but total volume and V40 volume did not. The reason was probably that V40 ratio is a more sensitive predictor and it reflects the interplay between changing total volume and V40 volume. For example, a thin patient’s total small bowel volume is smaller than that of patients with normal body weight. Although he has small V40 volume, his V40 ratio will increase and he is more likely to suffer from chronic GI toxicity.

The results of our univariate analyses showed concurrent chemotherapy was significantly associated with grade 1–2 GI complications. The percentage of grade 1–2 toxicity in patients with and without concurrent chemotherapy were 46% and 20%, respectively (*P* = 0.02). But it was not an independent predictors of GI complications in multivariate analysis. Mundt also gained the similar result, they reported that 53% gynecology patients who treated with IMRT received chemotherapy (cisplatin 40 mg/m^2^/week), no significant correlation was seen between the development of chronic GI toxicity and concurrent chemotherapy [24]. With regard to chemotherapy regimens, Pu found that in high-risk early stage cervical cancer patients, the incidence of late side radiation effects was similar between docetaxel/cisplatin group and single agent cisplatin group [25]. Our study also got the same result, docetaxel group and cisplatin group had no statistical difference in the incidence of late small bowel complications.

Several previous studies reported that patients with smoking, diabetes mellitus and lower BMI (<18.5 kg/m^2^) were more likely to experience worse GI toxicities after RT for gynecologic malignancies [15–19]. However, these factors cannot be found to be associated with chronic GI complications in our study. In general, the reasons are as follows: only a few Chinese females were addicted to smoking and suffered from diabetes mellitus at the mean age of 48 [26, 27]. Moreover, the value of BMI of the cervical cancer patients in our studies was 25 ± 4 kg/m^2^, higher than 18.5 kg/m^2^.

## Conclusions

We conclude that body position and RT total dose are significantly associated with grade 1–2 GI complications after postoperative IMRT in early-stage cervical cancer patients. maximum dose and V40 ratio of small bowel loops should be considered synthetically before radiation therapy. The optimal threshold were 5586 cGy (maximum dose), 28% (V40 ratio) of the small bowel loops.
